# Fatigue in patients with Juvenile Idiopathic Arthritis: relationship to perceived health, physical health, self-efficacy, and participation

**DOI:** 10.1186/s12969-016-0125-1

**Published:** 2016-12-06

**Authors:** Wineke Armbrust, Otto H. T. M. Lelieveld, Jolanda Tuinstra, Nico M. Wulffraat, G. J. F. Joyce Bos, Jeannette Cappon, Marion A. J. van Rossum, Pieter J. J. Sauer, Mariët Hagedoorn

**Affiliations:** 1 Department of Pediatric Rheumatology, University of Groningen, University Medical Center Groningen, Beatrix Children’s Hospital, Groningen, The Netherlands; 2 University of Groningen, University Medical Center Groningen, Center for Rehabilitation, Groningen, The Netherlands; 3 Department of Health Sciences, University of Groningen, University Medical Center Groningen, Groningen, The Netherlands; 4 Department of Pediatric Immunology, University Medical Center Utrecht, Wilhelmina Children’s Hospital, Utrecht, The Netherlands; 5 Reade, Center for Rehabilitation and Rheumatology, location: Dr. Jan van Breemenstraat, Amsterdam, The Netherlands; 6 University of Groningen, University Medical Center Groningen, Beatrix Children’s Hospital, Groningen, The Netherlands

**Keywords:** Juvenile idiopathic arthritis, Fatigue, Self-efficacy, Physical activity, Exercise capacity, Patient reported outcome, Participation

## Abstract

**Background:**

Fatigue is common in patients with JIA and affects daily life negatively. We assessed the presence and severity of fatigue in patients with JIA, including factors presumed associated with fatigue (e.g., disease activity, disability, pain, physical activity, exercise capacity, and self-efficacy), and whether fatigue is related to participation in physical education classes, school attendance, and sports frequency.

**Methods:**

The current study used baseline data of 80 patients with JIA (age 8–13) who participated in an intervention aimed at promoting physical activity. Primary outcome measurements were fatigue, assessed using the Pediatric-Quality-of-Life-Inventory (PedsQl)-Fatigue-scale and energy level assessed using a VAS scale. Other outcome measurements were disease activity (VAS Physician Global Assessment Scale), disability (Childhood Health Assessment Questionnaire), physical activity (accelerometer), exercise capacity (Bruce treadmill test), self-efficacy (Childhood Arthritis Self-Efficacy Scale), and participation (self-report).

**Results:**

Sixty percent of patients with JIA suffered from daily low-energy levels; 27% suffered from very low-energy levels more than half the week. Low energy levels were best predicted by disability and low physical activity. Fatigue measured with the PEDsQL was higher compared to the control-population. Disability and low self-efficacy were main predictors of fatigue. Self-efficacy was a predictor of fatigue but did not act as moderator. Fatigue was a predictor for sports frequency but not for school attendance.

**Conclusion:**

Fatigue is a significant problem for JIA patients. Interventions aimed at reducing perceived disability, stimulating physical activity, and enhancing self-efficacy might reduce fatigue and thereby enhance participation.

**Trial registration:**

Trial number ISRCTN92733069

## Background

Chronic auto-immune diseases such as juvenile idiopathic arthritis (JIA) are associated with impaired (social) functioning [1]. Fatigue is one of the most frequent complaints in JIA patients and identified as one of the causes behind impaired functioning. The majority of JIA patients (60-76%) report at least some fatigue; 15-19% of these report moderate to severe fatigue [2, 3]. Fatigue is not only present in young patients but extends into adulthood, even when the disease is extinguished [4]. Studies regarding the background of fatigue in JIA patients are limited. Interpretation of these studies is complicated through a lack of a clear definition of fatigue and the various measurements used [5]. The definition of fatigue in chronic diseases is often referred to as “*a persistent, overwhelming sense of tiredness, weakness or exhaustion, resulting in a decreased capacity of physical and/or mental work and is unrelieved by sleep or rest”* [6, 7]. This description implies that fatigue is a subjective feeling, leading to consequences in several domains. Fatigue is multidimensional, meaning that it can be physical or mental in its manifestation and cause. To provide information about these dimensions, multidimensional measurements are more appropriate for studying fatigue than unidimensional measurements [6, 8]. Unidimensional measurements, for example, a Visual Analogue Scale (VAS), can be used to assess the presence and the intensity of fatigue in an easy way, but does not provide information about the mental and physical dimensions [8].

Fatigue in JIA patients is a complex interplay between many factors, which has not as yet been fully revealed [5]. Fatigue cannot be entirely explained by disease-related factors, such as disease activity or use of medication. High levels of fatigue are also found in patients with inactive disease [9–11]; data on the effect of medication on fatigue are conflicting [12, 13]. A clear association has been found between fatigue and factors influencing perceived health, such as pain and physical disability [5, 11, 14]. Physical activity and exercise capacity are impaired in patients with JIA [15–18] but the extent to which these factors contribute to fatigue has not been studied. Data concerning the consequences of fatigue suggest that school attendance and participation in leisure activities are negatively affected by JIA [11, 19, 20]. One study showed normal school attendance [21]. Self-efficacy could play a role in dealing with symptoms associated with JIA. Self-efficacy refers to “*beliefs in one’s capabilities to organize and execute the course of action required to produce given attainments*” [22]. In adults with Rheumatoid Arthritis (RA) higher self-efficacy was related to lower levels of fatigue [23]. In children with JIA, the role of self-efficacy in the perception of disease-related factors, like fatigue, has not been studied [24]. To be able to intervene in patients’ experience of fatigue, it is necessary to understand the causes and consequences of it. It is also important to investigate the interplay of the various factors presumed related to fatigue.

The first aim of this study is to assess both the presence and severity of fatigue in JIA patients, along with factors that might be related to fatigue. The second aim is to study whether disease-related factors (medication and disease activity), perceived health (disability and pain) and physical health (exercise capacity, and physical activity) are independently associated with fatigue, and whether self-efficacy is also independently associated or acts as a moderator in this model. The third aim is to study whether levels of fatigue are related to participation in physical education classes, school attendance, and sports frequency, above and beyond disease-related factors, perceived health, and exercise capacity.

## Methods

### Patients

The current study includes JIA patients, age 8–13, who participated in an intervention study to improve physical activity (PA), called Rheumates@work (R@W Trial number ISRCTN92733069) [25]. Patients were recruited, between January 2011 and September 2012, from three pediatric rheumatology departments in the Netherlands: Beatrix Children’s Hospital of the University Medical Center Groningen; Wilhelmina Children’s Hospital of the University Medical Center Utrecht; and the Reade Center for Rehabilitation and Rheumatology in Amsterdam. Three hundred and eight patients were eligible for R@W and invited by letter; 83 were willing to participate. Finally, 80 patients were included [26]. Approval of the Medical Ethical Committee was obtained in each center. Inclusion criteria were: diagnosis of JIA according to the ILAR criteria [27], good comprehension of the Dutch language, and availability of a computer with Internet connection. Exclusion criteria were a physical disability that was caused by a condition other than JIA and that limited motor and/or exercise performance (e.g. paralysis or heart conditions), receiving cognitive behavioral therapy, and a disease activity defined as a Physician Global Assessment >2 cm out of 0 to 10. The current cross-sectional study analyzes the baseline measurements that were all collected at the same moment (or during the week before, i.e., energy level and PAL)

### Primary outcome measurements are fatigue and energy level

The *presence of fatigue* was measured with the Pediatric-Quality-of Life-Inventory Multidimensional Fatigue Scale (PedsQl-MFS) for children aged 8–12 years. The PedsQl-MFS includes 3 subscales: general fatigue, sleep/rest fatigue, and cognitive fatigue. Patients rated how often a symptom occurred during the past month, using a 5-point Likert scale (0 = never a problem, 1 = almost never a problem, 2 = sometimes a problem, 3 = often a problem, 4 = always a problem). Scoring was performed according to the manual, resulting in subscale and total scores between 0–100 (http://www.pedsql.org/). Higher scores indicate lower levels of fatigue. The correlations between the subscales and the total score of the PEDsQl-fatigue were high (r’s ranging from .72 to .86), suggesting that the total score properly reflected the subscales and thereby the different dimensions. Therefore, the total score was used to describe the presence of fatigue (Crohnbach’s α was .72). For ease of interpretation, correlation and regression analyses were performed with a reversed total score (fatigue_R_; 100-PEDSQoL). Higher scores on the fatigue_R_ indicate higher levels of fatigue.

*Energy level* was estimated using a unidimensional VAS score from 0–10. Zero meant no energy (for the patients: an “empty battery”) and 10 meant maximum energy (“battery fully charged”). Patients rated their energy level 3 times a day – morning, afternoon, and evening – for 7 days. Mean daily energy levels were calculated, as well as mean energy levels morning, afternoon, and evening. Low mean daily energy levels were defined as a VAS score of 8 and less, and very low energy as 5 and less. The number of days out of 7 days, with a low and very low mean daily energy level, was counted for each patient. Zero days meant a patient indicated no day with (very) low energy levels, whereas 7 meant the patient indicated a maximum number of days with (very) low energy levels.

Other measurements were divided into predictors of fatigue (disease-related factors, perceived health, and physical health), consequences of fatigue, and self-efficacy.

### Predictors of fatigue

#### Disease-related factors

*Patient characteristics*, such as type of JIA, disease onset, and medication (yes/no), were obtained from medical charts.

*Disease activity* was measured by a pediatric rheumatologist, expressed in a VAS Physician Global Assessment (VAS-PGA) anchored by 0–10 cm (0 = no disease activity to 10 = high disease activity).

#### Perceived Health

*Disability* was measured using the Dutch version of the Childhood Health Assessment Questionnaire (C-HAQ38) [28], which measured functional impairment in 9 domains. Scores ranged from 0 to 3, where 0 stands for no impairment and 3 for maximum impairment.

*Pain* was measured on a 0–10 cm VAS. Zero meant no pain, whereas 10 meant the maximum pain imaginable.

#### Physical health

*Physical Activity Level (PAL)*. Physical activity was measured using an accelerometer (Actical; Phillips-Respironics, Oregon, USA), which patients wore for seven consecutive days [29]. The omnidirectional accelerometer measures accelerations in any plane of movement, translates them into activity counts per time unit and to activity-related energy expenditure (AEE) in Kcal per day. PAL was computed by the formula: ([AEE * 4.1868] / [1000 + BMR] / 0.9.)/ BMR. Basic Metabolic Rate (BMR) rate was computed according to Schofield [30]. The wearing-time of the Actical on a day during the week had to amount to at least 8 h and on a weekend-days 6 h in order to count as a valid measurement [31]. This method has been described more extensively elsewhere [26]

*Exercise capacity (EC)* was measured on a treadmill by means of the Bruce protocol, and it was expressed as maximum endurance time [32, 33]. During the test, participants were vigorously encouraged to reach maximum exertion (as measured with a polar chest-belt), defined as a maximum heartbeat of 180 or higher. The test was terminated at the patient’s request or at the observer’s discretion.

#### Consequences of fatigue

Participation in school and physical education class were queried, with a 3-month recall period. *School attendance* was defined as no absence from school or absence for 1 or more days, related to the disease but not to infections or regular hospital visits.

*Participation in physical education class* was rated as complete, when the lessons were not adapted or when the child did not miss a class for reasons related to the disease.

*Sports frequency* (mean hours per week) was rated for the previous 3 months.

#### Self-efficacy

*Self-efficacy* was measured with the Childhood Arthritis Self-Efficacy Scale (CASE). The CASE includes 11 questions concerning capacity to manage JIA, on 3 different subscales: symptoms, emotions, and activity. Patients were asked, on a 5-point Likert scale, how certain they were to be able to manage various issues. One means very uncertain, and 5 very certain. Scores were standardized to compare the subscales [24]. For correlations and regression analyses, we used the symptom scale (self-efficacy_SYM_) and activity scale (self-efficacy_ACT_). This choice was based on the content of these scales, which corresponds best to the outcome measurements, that is, fatigue, disease related factors, perceived health, physical health and consequences of fatigue. Higher scores mean higher self-efficacy. Self-efficacy subscales correlated moderate to good (r’s ranged from .47 to .71), and Crohnbach’s α’s varied between .83 and .89.

Energy level and PAL were assessed during the week prior to the completion of a questionnaire that included the other predictor and outcome variables and the establishment of patient characteristics (via medical charts), exercise capacity and disease activity (via pediatric rheumatologist).

### Statistics

Statistical analysis was performed using SPSS-IBM statistics version 22. Spearman correlations were performed to study the correlation between gender, age, disease activity, use of medication, pain, disability, exercise capacity, physical activity level, and self-efficacy, on the one hand, and fatigue_R_ and mean daily energy level, on the other. All factors that were correlated *p* < .10 with fatigue or energy level were studied in a multivariate model. A moderating effect for self-efficacy was studied with interaction terms of the factors that appeared as significant predictors in the multivariate model.

Participation in physical education class, school attendance, and sports frequency was correlated with disease activity, use of medication, pain, functional ability, fatigue_R_, exercise capacity, and self-efficacy (Spearman). Energy level was not included, since it was considerably correlated with fatigue, and because the measurement of energy level is based on one week, while participation reflects the previous 3 months. Therefore, fatigue was considered as a more appropriate predictor. Correlates with a *p* value < .10 were included in a logistic regression analysis to predict school attendance and participation in physical education class, and in a multilevel regression analysis to predict sports frequency.

In the logistic regression model and in the multivariate model, values centered around the mean of the continuous variables were used. Residuals were checked for normal distribution. A p value of ≤0.05 was considered significant.

## Results

The sample included 80 patients, majority girls (65%), with a median age of 9.8 years (Table 1). Median disease activity on a VAS-PGA was low (0.3 cm) and 35% of the patient had a VAS-PGA of 0. The median disease duration was 2.9 years, and 75% of the patients were on medication. The total fatigue score was 77.8. The mean energy level was comparable in the morning (5.6 cm) and evening (5.5 cm), and highest in the afternoon (7.2 cm). Low mean daily energy levels were reported by 60% of the patients on all 7 days and by 78% on more than half the week. Seven percent of the patients reported very low levels of energy on all 7 days of the week and 29% on more than half the week (Table 1).

**Table 1:** Patient characteristics and outcome measurements of 80 patients with JIA

Characteristic	M *N*	[IQR] / (%)
Age	9.8	[8.7; 11.0]
Female	*52*	(65)
JIA classification		
-Oligo-JIA, persistent	*25*	(31)
-Oligo-JIA, extended	*11*	(14)
-Poly-JIA, Rf-	*25*	(31)
-Poly-JIA, Rf+	*3*	(4)
-Psoriasis related JIA	*4*	(5)
-Enthesitis related JIA	*3*	(4)
-S-JIA	*9*	(11)
Disease duration (yr)	2.95	[1.22; 6.19]
Disease activity (cm)	.30	[.00; .90]
On medication	*60*	(75)
Disability	.33	[.11; .75]
Pain (cm)	1.50	[.20; 3.85]
Fatigue	77.8	[68.1; 86.1]
Energy level (cm)		
- Mean morning	5.55	[4.04; 8.14]
- Mean afternoon	7.21	[5.73; 8.39]
- Mean evening	5.53	[4.21; 7.57]
- Mean daily	6.00	[4.92; 7.49]
Number of days low/ very low energy (*n* = 72)		
- 0	*7/25*	(10/35)
- 1-3	*9/27*	(13/38)
- 4-6	*13/16*	(18/22)
- 7	*43/5*	(60/7)
Exercise capacity	592	[489; 638]
PAL	1.53	[1.46; 1.60]
Participation PEC		
-Full	*53*	(66)
-Partial	*22*	(28)
-No	*5*	(6)
Full school attendance	*56*	(70)
Sports frequency/week	1	[.25; 3.00]
Self-efficacy		
-Symptom	3.75	[2.50; 6.72]
-Activity	6.88	[4.38; 9.38]

*M* Median, *IQR* Inter Quartile Range, *N* Number, *Rf* Rheumatoid factor, *yr* years, *cm* centimeters, *PAL* Physical Activity Level, *PEC* Physical Education Class

Seventy percent of patients reported full school attendance without absenteeism due to the disease. Full participation in physical exercise classes was seen in 66%, whereas the median sports frequency in free time was 1 h per week (Table 1).

High fatigue was correlated with low energy levels (Table 2). Patients who reported higher levels of fatigue showed higher disability, higher pain, lower EC, lower PAL, and lower self-efficacy scores. Fatigue was not correlated with use of medication, age, and gender, or with disease activity. High energy levels were correlated with low disability, low pain, a higher PAL, and being off medication.

**Table 2:** Correlations with total fatigue_R_ and energy level_MEAN_

	Total fatigue_R_		Mean daily energy level	
	R	p	R	p
Gender	.17	.13	-.22	.06
Age	.04	.71	-.15	.18
Disease activity	.08	.46	-.21	.06
Medication use	.12	.29	-.25	.03
Disability	.47	< .01	-.41	< .01
Pain	.38	< .01	-.35	< .01
Energy level_MEAN_	-.52	<.01		
EC	-.22	.05	.20	.07
PAL	-.30	<.01	.36	<.01
Self efficacy_SYMPTOM_	-.25	.02	.12	.30
Self efficacy_ACTIVITY_	-.44	<.01	.19	.10

*EC* Exercise capacity, *PAL* Physical activity, Gender 1 = male; Medication use: 1 = yes

In a multivariate model high disability and low self-efficacy_ACT_ predicted higher levels of fatigue, a lower PAL approached significance as a predictor of higher fatigue. The factors that were studied in this multivariate model (Table 3) explained 32% of the variance in fatigue. Self-efficacy_ACT_ was an independent predictor and not a moderator of the prediction of fatigue (Appendix 1). In a second multivariate model, high PAL and low disability predicted higher energy levels. The factors that were studied in this model explained 34% of the variance in the energy level (Table 3)*.*

**Table 3:** Prediction models for fatigue_R_ and energy-level_MEAN_

	Fatigue_R_^a^			Energy-level_MEAN_^b^		
	B	p	CI	B	p	CI
Estimate	.12	.10	−2.34; 2.58	7.48	<.01	6.07; 8.91
Gender				-.57	.14	−1.31; .18
Disease act_C_				-.30	.21	-.78; .17
Medication				-.43	.32	−1.26; .41
Pain_c_	.05	.94	−1.16; 1.26	-.00	.96	-.18; .17
Disability_c_	8.72	.01	2.00; 15.4	−1.2	<.01	−2.15; −.33
EC_c_	-.00	.73	-.03; .02	.00	.39	-.00; .01
PAL_c_	−24.5	.06	50.0; 1.05	4.8	<.01	1.32; 8.33
SE_ACTIVITYc_	−1.16	.04	−2.24; −.08			
SE_SYMPTOMc_	-.28	.58	−1.28; .72			

^a^R^2^ = .32; ^b^R^2^ = .30; *EC*_*c*_ Exercise capacity, *PAL* Physical activity, *SE*_*ACT*_ Self-efficacy Activity Scale, *SE*_*SYM*_ Self-efficacy Symptom Scale, _*c*_ Centered values; Gender 1 = male; Medication 1 = use of medication

Higher sports frequency was significantly correlated with lower fatigue, whereas correlations of fatigue with full participation in physical education class and school attendance approached significance (Table 4). High self-efficacy correlated significantly with participation in physical education class. The use of medication, disease activity, pain, and disability correlated with both school attendance and participation in physical education class (Table 4). A multivariate regression model revealed younger age, off medication, lower disability, less pain, higher fatigue, and higher exercise capacity as significant predictors of full participation in physical education class. The factors that were studied in this model predicted 82% of the variance (Table 5). With respect to school attendance, only pain reached significance, predicting 46% of the variance. Sports frequency was predicted solely by low fatigue, predicting 10% of the variance.

**Table 4:** Correlations with Total Participation in PEC, school attendance, and sports frequency

	School attendance		Full participation PEC		Sports frequency	
	R	p	R	p	R	p
Gender	.15	.19	-.19	.09	-.15	.20
Age	-.22	.05	-.20	.08	-.06	.57
Disease activity	-.22	.05	-.40	<.01	-.15	.18
Medication use	-.38	<.01	-.35	<.01	-.19	.08
Disability	-.29	<.01	-.58	<.01	-.19	.10
Pain	-.37	<.01	-.48	<.01	-.15	.18
Fatigue_R_	-.20	.07	-.20	.07	-.30	<.01
EC	-.04	.74	.32	<.01	.11	.34
Self efficacy_SYM_	.11	.34	.27	.02	.14	.23
Self efficacy_ACT_	.11	.34	.26	.02	.03	.79

*PEC* Physical Education Class, *PAL* Physical Activity Level

**Table 5:** Prediction models for participation in PEC, school attendance, and sports frequency

	School attendance^a^			Full participation PEC^b^			Sports frequency^c^		
	B	p	SE	B	p	SE	B	p	95%CI
Gender				−1.83	.15	1.26			
Age	-.35	.11	.22	−1.29	.04	.64			
Dis act	-.06	.87	.37	−1.53	.06	.81			
Medication	-.20.4	1.0	8.6x10^3^	−3.31	.04	1.62	-.43	.21	−1.13; .25
Disab. _c_	-.13	.88	.85	−4.90	.02	2.06			
Pain_c_	-.32	.02	.14	-.69	.02	.29			
Fatigue_Rc_	-.09	.75	.03	.14	.04	.07	-.03	.01	-.05; −.01
EC_c_				.02	.03	.01			
SE_SYMpTOMc_				.03	.25	1.34			
SE_ACTIVITYc_				.12	.15	2.05			
Est	21.0	.99	8.6x10^3^	4.34	.01	1.58	1.96	<.01	1.37;2.56

^a^R^2^ Nagelkerke .46 (logistic regression); ^b^R^2^Nagelkerke .82 (logistic regression); ^c^R^2^ = .10 Multilevel regression analysis. Participation: 1 = total participation; School attendance, 1 = full attendance during the previous 3 months; gender: 1 = male; medication: 1 = yes; dis act = disease activity; *diasb.* disability, *fatigue*_*R*_ reversed fatigue scale, *EC* exercise capacity. *SE* self-efficacy, *est* estimate, _*c*_ centered values

## Discussion

In this study, we evaluated the presence and severity of fatigue in children with JIA and studied factors that might be related to fatigue. This is the first study that has investigated the associations between fatigue and self-efficacy, exercise capacity, and physical activity in this group. In addition, the potential consequences of fatigue, such as less participation in physical exercise classes, poorer school attendance, and sports frequency, were also studied.

Results of this study showed that patients indicated slightly higher fatigue levels (77.8) compared to a historical control-population (80.5) [34] and to a Dutch sample (78.7) [35]. Both differences were not significant. However, this Dutch sample of children included 11% patients with chronic conditions, who indicated significantly higher fatigue scores compared to the healthy subjects in that cohort [35]. Patients in this Dutch cohort were older (11.4 years) than the patients in this study. As it is known that (pre) pubertal children have higher levels of fatigue some caution is needed to compare these groups. In comparison to our findings, other studies on fatigue in JIA patients have shown lower levels of total fatigue [9, 36].

The results of this study showed that more than a quarter of the patients reported very low energy levels more than half the days per week. This finding corresponds with results of a recent study, where severe fatigue was found in 25% of adolescents with JIA [11], indicating that fatigue is a serious problem in JIA patients. The “battery” was only charged to a maximum of 72% in all the patients and the energy levels varied during the day; patients felt fittest in the afternoon. Bromberg and colleagues found the same daily variation of levels of fatigue measured with a VAS fatigue [13]. These results imply that important activities should be scheduled in the afternoon.

We found that both uni- and multidimensional measurements can be used to determine the level of fatigue. However, related factors were different for both measurements, as single correlates as well as in a prediction model, indicating that they are not interchangeable. The most striking difference in the prediction models was that, after adjustment for other factors, self-efficacy was associated with fatigue, while not with energy level. An explanation might be that the items of the PEDsQL include the impact of fatigue, for example, “I feel too tired to do the things I like to do” [34]. Probably self-efficacy plays a role in the patient’s perceptions of the consequences of fatigue in daily life, while the VAS only indicates the intensity of fatigue at that moment. The choice between both measurements depends on what purpose is aimed for. When underlying mechanisms and consequences of fatigue on more dimensions are studied, the use of a multidimensional scale like the PEDsQL is preferred. For clinical use, a VAS has the advantage of reporting the intensity and frequency of fatigue or energy levels, and the results can be interpreted more easily. Combining the uni- and multidimensional measurements provide the most complete information.

Fatigue and energy levels were not related to disease-related factors, which corresponds with previous studies [9–11]. In all these studies, disease activity was low, as it was in our study, which might have affected the outcome.

Disability was correlated with both fatigue and low energy levels, when adjusted for other factors. The relationship between disability and fatigue is well known [9, 36], although the exact mechanism is unclear. Disability, as perceived by the patient, is not related to disease activity; it is underestimated in cases of active disease, though overestimated as the level of pain increases [37, 38]. A possible explanation for this is that, in cases of active inflammation, the child accepts the physical impairments and adjusts his/her goals, thereby experiencing the disability as less than expected. During remission, children strive for the same physical achievement as healthy peers, which is not always realistic. This can result in a feeling of disability and also fatigue because of high physical demands and emotional disappointment at not achieving goals.

Pain was correlated with fatigue and energy levels; however, in our prediction models, pain did not retain significance, which contrasts with results from other studies [5, 11], Pain, in general, is not correlated with disease activity, whereas it is associated with disability and psychosocial factors [39]. All these factors also correlate with fatigue [5], suggesting a cluster of correlated symptoms. In our population, pain was rather low, which might explain the lack of significance for pain in the prediction model. Low pain scores might have resulted from selection bias due to choosing patients willing to participate in a program promoting physical activity. Another reason why pain seemed to be less important might be the younger age of our patients: younger JIA patients indicate less pain compared to adults with RA [11, 40].

High PAL was significantly associated with high energy levels in the model, when adjusted for other factors, whereas PAL almost reached significance in the prediction model for low fatigue. In children, this relationship has not been reported previously, but our finding corresponds with results found in adults with RA [41, 42]. The relationship between PAL and fatigue in RA patients appeared to be bidirectional; low PAL resulted in fatigue, and fatigue acted as a barrier to being active [41, 42]. Exercise capacity was correlated with fatigue but did not retain significance in the prediction model, indicating that factors other than exercise capacity play a role in the feeling of fatigue. This is also seen in patients with chronic fatigue syndrome, in which exercise capacity is only reduced in a minority of patients [43].

High self-efficacy for activities was associated with low fatigue, when adjusted for other factors. In JIA patients, the association between self-efficacy and fatigue has not been previously studied. The role of self-efficacy was established as a factor for predicting pain in JIA patients [39]. This finding, combined with our finding that self-efficacy is not a moderator but an independent predictor, might indicate that self-efficacy plays an important role in the perception of disease-related factors in JIA patients. Some patients feel severely restricted in their life as a result of pain and fatigue while others, with the same disease severity, live an unconcerned life. Self-efficacy could play a role in patient’s resilience in adjusting to the disease and the accompanying symptoms. Our findings suggest that self-efficacy might be an interesting target in JIA patients’ daily care and also for interventions for improving outcomes regarding perceived disability, pain, fatigue, and, in turn, participation. Fatigue, for example, can act as a barrier to being active; however, being active can lead to lower fatigue [42]. By improving self-efficacy vis-à-vis fatigue, deploying activities and participation, this vicious circle can be broken. An example of such strategy is Pender’s health-promotion model, which includes education and self-efficacy strategies to overcome barriers from certain behaviors in order to benefit from newly learned ones [44].

Low fatigue correlated with full participation in physical education class. However, in the prediction model, high fatigue appeared to be associated with full participation, which is inconsistent with our expectations. A reason could be that factors other than fatigue, for example, pain and perceived disability, are decisive in whether or not to participate. It could also be that physical education class results in fatigue, because the classes are obligatory, and the incentive to perform at the same level as healthy peers is high.

Fatigue was a predictor of lower sports frequency. Other studies suggested fatigue as one of the determinants for JIA patients participating less in leisure activities [20]. We believe that this correlation is based on the same assumption as the correlation between fatigue and PA. The feeling of fatigue can act as a barrier for participation in sports, whereas taking part in sports activities may lead to a decrease in fatigue in the long term. Fatigue was not a predictor of school attendance, which contradicts a recent paper showing that adolescents with high fatigue had poorer school attendance compared to those with lower fatigue [11]. However, other papers reported data consistent with results found in our study [45, 46].

The main limitation of our study is that patients were recruited from another study aimed at improving PA in children with low disease acitivy. This might have caused a bias in our sample in different ways. Patients who feel more fatigued could be more motivated to participate in an activity program to improve their fitness. In contrast, and maybe more likely, patients who feel fatigued do not have the energy to participate in an activity program. While the lack of insight in the possibility that a program like R@W could also relieve their fatigue could prevent them from participating. Only patients with low disease activity were included which could have caused a bias. Nevertheless, the level of disease activity in our sample is a fair reflection of the disease activity in the chronic JIA population, currently under treatment. We did not measure puberty, a factor known to contribute to fatigue. Another limitation is that participation in school, physical education class, and sports was a question directed to the patient, and not verified by schools or sports clubs, which might have led to an overestimation of participation. Finally we did not include sleep duration and or quality in this study. Little is known from the literature about the relation between sleep and fatigue in patients with JIA and the studies that are published show conflicting results [5].

## Conclusion

We conclude that, fatigue is a serious problem in JIA patients; measuring fatigue using uni- and multidimensional measurements provides complementary information concerning intensity, frequency, and predictors of fatigue. The finding that self-efficacy was strongly associated with fatigue, when adjusted for other factors, is important. This implies that enhancing self-efficacy strategies in JIA patients may improve outcomes regarding perceived health, including fatigue and physical health, thus resulting in greater physical activity and participation.

## Appendix 1

**Table 6:** Results of the multivariate analysis of Fatigue_R_ exploring interaction

	B	p	CI
Estimate	22.5	. < 01	19.8; 25.1
EC _c_	-.02	.14	-,05; .01
SE_ACTIVITY c_	−1.94	<.01	−3.0; −.94
EC _c_ *SE_ACTIVITY c_	,00	.49	-,01; .02
Estimate	22.6	<.01	80.0; 74,8
PAL _c_	−28.9	.04	−55.2; −1.93
SE_ACTIVITY c_	−1.85	<.01	−2.8; −.92
PAL _c_ *SE_ACTIVITY c_	-.99	.85	−11.6; 9.63
Estimate	22.4	<.01	19.8; 25.1
Pain _c_	1.45	.01	.42; 2.5
SE_SYMPTOMS c_	−1.26	.01	−2.2; −.35
Pain*SE_SYMPTOMS c_	-.07	.66	-.41; .26
Estimate	22.6	<.01	19.9; 25.2
Dysability _c_	10.9	<.01	5.3; 16.5
SE_SYMPTOMS c_	-.82	.09	−1.77; .13
Dysability _c_ *SE_SYMPTOMS c_	.27	.80	−1.85; 2.39

*EC* exercise capacity, *SE* self efficacy, _*c*_ centered values

* multiply
